# The “Healthy Akame!” community – government – university collaboration for health: a community-based participatory mixed-method approach to address health issue in rural Japan

**DOI:** 10.1186/s12913-020-05916-w

**Published:** 2020-11-30

**Authors:** Marinda Asiah Nuril Haya, Shuhei Ichikawa, Yukino Shibagaki, Akame Machijuu Genki Project Community Advisory Board, Hideki Wakabayashi, Yousuke Takemura

**Affiliations:** 1 Department of Family Medicine, Mie University Graduate School of Medicine, Edobashi 2-174, Tsu city, Mie prefecture Japan; 2 Department of Community Medicine, Faculty of Medicine Universitas Indonesia, Jl. Pegangsaan Timur no. 16, Central Jakarta, Indonesia; 3 Faculty of Clinical Medicine, Mie University Graduate School of Medicine, Edobashi 2-174, Tsu city, Mie prefecture Japan; 4 Community Integrated Support Center, Department of Welfare and Children Nabari City Office, Konodai 1-1, Nabari city, Mie prefecture Japan; 5 Akame Machijuu Genki Project Community Advisory Board, Akame Community Center, Jouroku 238-1, Akame region, Nabari city, Mie prefecture Japan; 6 Department of Community Medicine Kameyama, Mie University School of Medicine, Edobashi 2-174, Tsu city, Mie prefecture Japan; 7 Department of Family Medicine, Graduate School of Medical and Dental Sciences, Tokyo Medical and Dental University, Yushima 1-5-45, Bunkyo, Tokyo, Japan

**Keywords:** Health promotion, Community-based participatory research, Health needs assessment, Program development, Mixed-method, Rural health

## Abstract

**Background:**

Although Japan has a decentralized public health system, local governments have considered expert opinions over those of the community in decisions about public health programs. Differences in communities’ interests may create gaps between health program objectives and implementation. We hypothesized that community-based participatory research (CBPR), which involves the community at every step, promotes effective program implementation and community empowerment. This study addressed the first step of CBPR, assessing community needs and developing tailored health program for a rural community in Japan.

**Methods:**

In this sequential exploratory mixed-method study (qualitative followed by quantitative), we first formed a community advisory board (CAB) representing community organizations, city officials, and university researchers. The CAB conducted group discussions with community residents to identify the community’s health issues and strengths. These group discussions were analyzed using thematic analysis, and the results were used to develop a questionnaire, which was subsequently sent to all households in the community to obtain priority scores for health issues and proposed action and to assess willingness to participate in community health program. The CAB then designed a program using the overall study results.

**Results:**

Ten group discussions with 68 participants identified the following health issues: 1) diseases; 2) unhealthy behaviors; and 3) unsupportive environment. Nature, vacant lots, and local farms were considered local strengths. Of a total of 1470 households in the community, questionnaires were collected from 773 households. Cancer, lifestyle-related diseases, and cerebrovascular diseases were ranked as the most important health problems. Improving services and access to medical checkups, use of public space for exercise, local farming, and collaboration with the community health office were considered necessary to address these health problems. Considering feasibility and the availability of resources in the community, the CAB decided to focus on lifestyle-related diseases and designed activities centered on health awareness, nutrition, and exercise. These activities drew on community’s strengths and were adapted to Japanese culture.

**Conclusions:**

The community’s priority health problem was closely related to the epidemiology of diseases. The CBPR approach was useful for identifying community’s needs and for designing a unique community health program that made use of local strengths.

**Supplementary Information:**

The online version contains supplementary material available at 10.1186/s12913-020-05916-w.

## Background

As the country with the longest life expectancy in the world, Japan has a growing population of older adults and a rising incidence of non-communicable diseases, among other health problems. The longer life expectancy observed in Japan is accompanied by more older adults requiring long-term care, and the cost of providing this care is increasing each year [1]. Cancer is the leading cause of death in Japan, followed by other chronic diseases such as cardiovascular and cerebrovascular diseases [2].

Japan has adopted a decentralized public health system, with supervision by the Ministry of Health, Labour and Welfare, which decides the general direction for public health policy. On the basis of this overall guidance, municipal and local governments then prepare more specific targets and policies [3, 4]. In the private sector, companies are responsible for their workers’ health. Some public health policies for certain populations are regulated by different ministries; for example, policies for students’ health fall under the mandate of the Ministry of Education [3, 5].

In 2000, the Ministry of Health, Labour and Welfare launched the “Health Japan 21” strategy to promote risk prevention at the national level. The goals of this strategy are to reduce health inequality, prevent non-communicable diseases, and improve healthy life expectancy and quality of life in Japan [3, 6]. The first term (2000–2012) evaluation of “Health Japan 21” showed that only 17% of the strategy’s targets had been achieved, with improvements toward 47% of the targets, worsening in 15% of the target areas, and the rest remaining at the same level [3, 7]. The government has designed various programs such as cancer screening program and healthy ageing promotion projects. However, utilization of cancer screening remains low, and the participants in healthy ageing programs are mostly only older adults [8, 9], although the participation of younger people in such programs earlier in the life course is important for preventing chronic diseases and achieving healthy aging.

Because of Japan’s decentralized public health system, achievements vary across municipalities, with some performing well and others underperforming [3]. This discrepancy is especially high when comparing urban and rural areas of Japan. Rural regions make up more than 70% of Japan’s total area, but only around 20% of the country’s population are rural residents [10]. Through the process of urbanization, people in Japan—especially the younger generation—have moved to the large cities, leaving rural areas with smaller populations and a higher proportion of residents who are older adults [11]. This situation has driven an unequal distribution of physicians, as well as other health care service providers [12, 13]. Fewer facilities, less human resources, and greater difficulty in accessing health care services have compromised people’s health in rural areas of Japan. A previous study has shown that older people in rural Japan were less healthy compared with their counterparts living in urban areas [14].

In Mie Prefecture, located in central Japan, the majority of the population resides in industrial areas, and the half of the prefecture’s area that is rural is much less populated. Mie Prefecture is among the many prefectures in Japan with a less tailored program in place for the “Health Japan 21” strategy [15]. Many of Mie Prefecture’s health promotion programs simply adopt the actions designed by the Ministry of Health, Labour and Welfare at the national level, which were developed largely on the basis of expert opinion rather than using community input. This top-down approach to program design has created one-size-fits-all programs, and different priorities across communities may create gaps between the objectives and implementation of these health programs, resulting in low uptake of these programs at community level. A survey conducted in 2010 showed that a perceived lack of fairness and insufficient public participation in decision making were the main reasons for dissatisfaction with the healthcare system among Japanese people [16].

To complement the government’s top-down program, a program with a bottom-up approach could fill the program implementation gap at community level, enabling public health programs and community interventions to be conducted more effectively. However, reports are lacking on this type of bottom-up program, with a participatory approach seeking to clarify and address community needs in Japan.

Previous studies have used the community-based participatory research (CBPR) approach to identify community health needs [17, 18] or to understand communities’ perceptions regarding a certain health issue [19]. In conjunction with the process of identifying community needs, researchers could also use the CBPR approach to evaluate the potential challenges as well as strengths in a particular community. We considered the implementation of CBPR suitable for our study purpose. CBPR is a form of action research, in which the study is conducted *with* people, not *on* them. This approach involves community partners at every step, from assessing community needs to planning, executing, and evaluating the program. Therefore, the approach is a good fit for health promotion initiatives, which are supposed to enable people to take action, increase their control over the determinants of health, and improve their health [20]. In addition, the collaboration and equal partnership with the community in CBPR can create a sense of community ownership of the program, increasing the chances of the program’s sustainability.

To develop a program that is appropriate for a particular community, it is necessary to identify the priority health needs in the community. Therefore, in this first step of our CBPR project, we collaborated with a community and stakeholders in the area to assess the community’s health needs, identify the community’s strength, and develop a tailored program to address the community’s needs. The study described in this article is part of a larger project aiming to develop a community-based health program that is structured, evaluable, sustainable, and able to attract a broad audience in the community.

## Methods

### Study design

This study used CBPR, involving community partners throughout the entire process, from the planning of the intervention (hereafter referred to as the program) to its execution and evaluation, as well as a reflection on the intervention process [21]. In this article, we report the first part of the CBPR, which consisted of a community needs assessment and the development of a program. Within this approach, we applied sequential exploratory mixed method (a qualitative study followed by a quantitative study).

### Setting

This first step of this CBPR project took place from July 2017 to October 2018 in the Akame region of Nabari city. Nabari city is a small city with a population of 77,493 as of 2017 located in a rural area of central Japan [22]. It is one of the cities in Japan that has a “unique” health promotion program policy in contrast to the municipalities implementing the nationally designed one-size-fits-all health program. Nabari City hosts a separate community health office (*machi no hoken shitsu*) for each of the 15 regions in the city. Initially built to support long-term care in the community, these offices later developed into places of consultation for community members of all ages, offering advice on topics ranging from child health to caregiving issues for older adults. These offices also serve to support community health promotion and prevention activities [23].

In 2015, Nabari City also initiated a healthy community project (*machi juu genki*), recruiting volunteers to become community health leaders. In a city with a high proportion of older adults, this healthy community project aimed to promote a healthy lifestyle and to prevent the need for long-term care [24]. A part of the city’s health promotion budget is distributed to different community development organizations (*machi zukuri iinkai*) to support this project.

Under the healthy community project, each community was given the freedom to make decisions about their own project activities. There were no mechanisms in place for control or evaluation of how the budget was spent or for standardized evaluation of the health activities carried out in each community. Many of the activities were designed as multiple separate events, with no clear goal or objective evaluation method. The majority of the activities were conducted on the weekdays and were therefore attended mostly by older residents who had retired from work. Our research team from Mie University collaborated with Nabari City officials and community members to design a community-based health program that is more structured, evaluable, sustainable, and able to attract a broad audience in the community, compared with the existing project.

We looked for a potential community partner that engaged many community residents in community activities and that included a good representation of young people. After observing several communities and holding discussions with city officials, we approached the Akame region as a potential community partner. Akame is a small region in Nabari City, with a population of 3629 as of 2017. Older adults make up more than one-third of the region’s population [22]. It is one of the oldest regions in the city, dating back over 1000 years. The regional community maintains a 770-year-old intangible cultural heritage in relation to one of Japan’s largest and most historical Buddhist temple. The region is famous as a destination for natural tourism. In ancient times, people in the community mainly worked as farmers, and many residents continue to practice commercial or subsistence farming. Various activities are offered through the local community center, such as social, cooking, sports, and cultural clubs, which are mostly attended by older adults who have retired and thus have time for leisure activities. Younger community residents are active in the youth association. Prior to this project, the community development organization and the community health office in Akame conducted several health activities in the community. Some of these activities were held annually (e.g., the *Furusato* (hometown) Walk and checkups to assess bone mineral density or body composition). Others activities were offered every other month (e.g., square-stepping exercise classes) or irregularly (e.g., health education seminars).

### Community engagement

City officials introduced the university researchers working on this project to the identified potential partner community. First, the university researchers visited the community and held a meeting with community development organization representatives and staff members from the community health office. The community representatives, city officials, and university researchers agreed that an iterative process would be necessary to develop a sustainable program. After this initial meeting, the university researchers participated in health-related and social activities in the community to familiarize themselves with the community residents and to observe the community’s culture.

### Establishment of community advisory board (CAB)

To design and carry out the study, we established a community advisory board (CAB) that represented community organizations, city officials, and the university researchers. Community members were recruited by recommendation from community organization leaders. Fifteen community members were initially recruited through the community organizations, and an additional five community members were identified through community health leaders who were recruited and trained by the city. In addition to these community members, the other members of the CAB were two city officials who oversaw the community, two community health office staff members, two university researchers, and one physician from the city hospital, for a total of 27 CAB members. All CAB members had equal rights, and each played a role throughout the research and project development process.

The first and most significant challenge in this study was to achieve understanding among the CAB members regarding how CBPR differs from the usual research that they had previously encountered. The community residents thought of research as an academic activity that is exclusively owned and carried out by university researchers. Therefore, at the beginning of our study, we explained the CBPR approach to the CAB members. Ideally, in CBPR, there should be a transfer of knowledge regarding research from the academic partners to the community partners, and the community partners should be involved in creating the research proposal. However, the community partners in this study were very unfamiliar with the procedures related to research administration. To avoid creating an unnecessary burden for the community partners, we decided to divide our roles as follows. The university researchers were responsible for all procedural tasks necessary for the research. The community partners played the role of a bridge between the researchers and the community to recruit participants, gather data, and provide contextual insight into the findings. The study design, program planning, and all decisions affecting the program were discussed in CAB meetings.

### Data collection

Our target participants were adult (age 20 years or older) community residents who were capable of making decisions independently. First, we conducted a qualitative study to assess people’s perspectives about the health issues in the community, the community’s strengths, and proposed ways to solve the identified issues. In discussion with the CAB, it was proposed that holding a community forum (world café) [25] would be more time efficient than conducting a conventional group discussion. Because the community had already been holding these community forums for the past few years, community members were more familiar and comfortable with this method. We distributed flyers to all households in the community and also posted large posters on community bulletin boards to recruit men and women from various age groups as participants. Considering the human resources (facilitators) and space available, we set a limit of the first 80 persons who registered as participants in the world café. The facilitators were provided with a guide for the discussion one week before the world café, and this guide was discussed among the facilitators and university researchers to adjust difficult terms and decide on methods for delivering and following up on the questions in the guide. The guide included the following main questions to be asked during the world café:

1. What do you consider the main health issue/problem in the community?
2. What are the community’s resources and strengths that are beneficial for health? How do you think the community’s strengths can contribute to resolving these health issues?

In addition to these main questions, the guide also provided examples of follow-up questions and probes (Additional File 1). However, the follow-up questions and probes were flexible and could be changed depending on the facilitators’ judgment. The community forum participants were divided into 10 groups of six to seven people, each of which was facilitated by a CAB member. After the discussion, a representative from each group shared a summary of the group’s discussion with all forum participants. All group discussions were recorded and transcribed verbatim for qualitative analysis.

As the second step in the research, a quantitative questionnaire survey was conducted to score the importance of the identified health issues and proposed action. The questionnaire items were developed using the categories extracted from the analysis of the qualitative study described above. All items were assessed on a five-point Likert-type scale, ranging from *least important* (= 1) to *most important* (= 5). The self-administered questionnaires were distributed to all households in the community and collected anonymously. We also gathered data about respondents’ preventive behaviors; past participation in community activities; community identity and commitment to the community, which will be explored in another article; and willingness to participate in a community health promotion program. Representatives of Akame’s sub-regions helped to distribute and collect the questionnaires.

### Analysis

The qualitative data were analyzed using thematic analysis [26]. The initial qualitative analysis was conducted with NVivo 10 software [27] to extract keywords, generate codes, and categorize the data into themes. The first and second authors then re-visited the data to check the codes, categories, and themes to make connections among the identified themes. The themes, categories, and example discussion extracts are presented in Table 1 for better understanding.

**Table 1:** Perspective on health issues, community strengths, and proposed solutions

Theme	Sub-theme	Categories	Quotes
Goal	Staying independent until death		*Our deepest wish is pin-pin-korori (to die suddenly and painlessly after living a long and healthy life)*
Health Issues	Diseases	− Cancer − Cerebrovascular diseases − Life-style related diseases (metabolic syndrome, hypertension, diabetes mellitus, obesity) − Cardiovascular disease − Musculoskeletal diseases/ illness − Mental health problems	*There are many cancer survivors. What I fear is the fact that some people died from cancer. They died suddenly immediately after their retirement...* *Our issues were lifestyle disease, lack of place for exercise and lack of venue for community’s communication.* *Building up stress is most damaging to your health*
	Unhealthy behaviors	− Smoking − Alcohol drinking − Unhealthy diet − Sedentary lifestyle − Low participation in medical checkup and screening	*You keep smoking although it is not good for health.* *Now, lifestyle is westernized prefers meats a lot, it is fatty with accumulated cholesterol.* *… You don’t see people walking outside, like someone who mentioned lack of exercise earlier.*
	Unsupportive environment	− Lack of places to exercise − Costly vegetables − Lack of medical facility that offer comprehensive checkup − Difficulty to access medical facilities − Lack in information dissemination	*We have neither playground nor community for people that help us exercise* *Vegetable prices sharply rose and leafy vegetables do not last long. We do not eat much vegetables* *Transportation to the hospital is not convenient although there are more and more older people*
Community’s strength	Nature	− Mountain area, waterfall, etc. − Fertile land	*Akame is rich in nature, we should make better use of it to improve our mental and physical health*
	Network	− Community health office − Sports and social clubs	*I think our advantage is that we have Machi no hoken-shitsu*
	Communication platform	− Meet up events − Local newsletter	*We should make better use of facilities such as Fureai Café.*
Proposed solution	Improve health care services and access	− Services, facilities, and access for medical checkup − Community bus	*It is very troublesome to take older people to different hospitals for different check-ups. We need a program that offer comprehensive medical check-ups at one place for reduced cost.*
	Utilize local strength	− Public facility for exercise − Local farmers market and agriculture class	*I hope Yume Hiroba will be open to public regularly.* *Cooking practice using locally grown vegetables.*
	Strengthen community capacity	− Inter-generation communication − Role model and health leader	*Their horizons will be broadened if there is a place to frankly exchange information irrespective of age, generation and region, like we had today.*
	Facilitate behavior change	− Exercise/sports class and event − Information dissemination − Nutritional education and cooking class − Smoking cessation program	*We should ask each other to go for a walk.* *I want guidance on nutritional balance.* *If we can be connected with the information on daily basis, it would change our mind.*

For the quantitative data, descriptive statistics are presented for the questionnaire respondents’ sociodemographic characteristics, health issues priority score, proposed action score, preventive behaviors, past participation in community health activities, community identity, commitment to the community, and willingness to participate in a community health program. We then used logistic regression analysis to examine the association between the predictor variables (sex, age group, educational background, preventive behaviors, past participation in community activities, community identity, and commitment to the community) and willingness to participate in a community health program. The logistic regression model with the lowest Akaike information criterion value, the lowest residual sum of squares, and the highest Nagelkerke pseudo-*R*^*2*^ was chosen as the best fitting model. Multicollinearity was assessed using the variance inflation factor. The statistical analysis was conducted using R, version 3.5.2 [28], with the readxl [29], psych [30], tidyverse [31], dplyr [32], DAAG [33], and BaylorEdPsych [34] packages. The CAB discussed both the quantitative and the qualitative results to identify the highest-priority health problem and to develop a one-year health program to address this problem.

## Results

### Qualitative results

The community forum was attended by 68 participants: 10 young adults (aged 20–40 years), 18 middle-aged people (aged 41–64 years), and 40 older adults (aged ≥65 years). More than half of the participants were women. Despite not being explicitly asked this question, the study participants talked about what “health” meant to them at the beginning of the discussion. When the participants discussed “health”, they shared a similar goal: to be able to live independently for as long as possible (longer healthy life expectancy).

The group discussions generated the following sub-themes for health issues: 1) diseases; 2) unhealthy habits; and 3) an unsupportive environment. In addition, mental health was also considered important, with participants mentioning aspects such as stress, depression, and dementia. The discussions also emphasized the importance of social connection and communication.

Nature was considered as a strength of the community with the potential to improve community members’ health. The research community is surrounded by beautiful forests, waterfalls, parks, and fertile land. The forum participants thought that these surrounding natural areas had the potential to be not only tourism spots, but also places to exercise (e.g., locations for walking and hiking). Many people in the community engaged in farming, from the household subsistence level to the large commercial scale. Engaging in farming activities was considered beneficial for keeping people physically active, and community members thought that older people could teach younger community residents how to perform these activities. Opening a local farmers’ market was proposed as a possibility for meeting the needs of community residents who did not engage in farming and wished to purchase vegetables at more affordable prices. This could also provide local farmers with a chance to earn money by selling their extra vegetables.

Existing community networks and communication platforms (e.g., *Fureai* salons or community cafés and a monthly community newsletter distributed to every household in the community) were considered the best and most practical options for disseminating health information. Collaboration with the community health office was also considered highly necessary for community health improvement. The community health office staff had been building bonds with the community for a long time, not only assisting with health activities in the community, but also participating in the community’s social activities such as primary school meetings, community center gatherings, and activities of the older adults’ club. The community health office also provided consultation on child health and long-term care services for older people, and even acted as the neighborhood *onee-chan* (older sister), listening to primary school and high school students’ worries and offering emotional support for people with mental health problems or cognitive impairment. Therefore, community members depended on the community health office staff members and had high expectations regarding their involvement in community health activities.

**Fig. 1:**
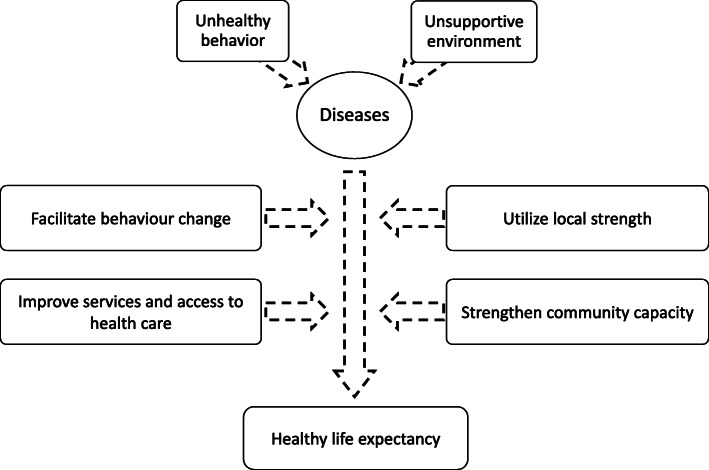
Relationship between health problems, proposed action, and goals in the community Unhealthy behaviors and an unsupportive environment were considered predisposing factors that contribute to diseases, which, in turn, affect community members’ healthy life expectancy. Improvement of services and access to medical checkups and screening, utilization of local strengths and potential, strengthening community capacity, and facilitating behavior change were thought to improve people’s health, resulting in a longer healthy life expectancy

In addition to improvements within the community, improvement in health services and access to health facilities for medical checkup were also considered important. Only a few clinics operated in the community, and none of these facilities offered comprehensive medical checkups or screening. To obtain various types of cancer screenings, the community residents had to travel to several different facilities. Because public transportation did not operate frequently enough in the area, access to healthcare facilities was difficult, especially for the older adults living in the community. The relationships between each of these themes is depicted in Fig. 1.

### Quantitative results

Of a total of 1470 households in the community, questionnaires were collected from 773 households. We categorized the participants into three age groups: young adults (aged 20–40 years), middle-aged (aged 41–64 years), and older adults (aged ≥65 years). The proportions of men and women were balanced in the sample. The majority of the respondents had graduated from high school or above and were currently employed. More than half of the respondents reported that they practiced preventive behaviors, with eating a healthy diet, attending medical checkups, and not smoking as the most frequently reported practice. More than half of the participants seldom or never attended health-related community activities. Details on the participants’ characteristics are described in Table 2.

**Table 2:** Participants’ characteristics, preventive behaviors, and community participation

Characteristic	Frequency (%)	Mean ± SD (range)
Age (years)		64.3 (20 to 93)
Sex		
Male	387 (50.0)	
Female	381 (49.3)	
Educational background		
Elementary – Junior high school	85 (11.0)	
High school	415 (53.7)	
Vocational College/ University	250 (32.3)	
Employment status		
Employed	396 (51.2)	
Unemployed	355 (45.9)	
Opinion on disease prevention		
Important	714 (92.3)	
Not important	7 (0.9)	
Do not know	53 (6.8)	
Preventive behaviors		
Exercise	369 (47.7)	
Healthy diet	417 (53.9)	
Vaccination	284 (36.7)	
Medical check-up	451 (58.3)	
Not smoking	455 (58.9)	
Not drinking alcohol	261 (33.8)	
Others	38 (4.9)	
Barriers to healthy lifestyle/ disease prevention		
Lack of time	138 (17.8)	
Lack of willingness	106 (13.7)	
No companion	45 (5.8)	
Lack of facility	66 (8.5)	
Do not how to start/ what to do	84 (10.9)	
Others	21 (2.7)	
Participation in community activities	89 (11.5)	
Frequent	119 (15.4)	
Seldom	430 (55.6)	
Rarely/ never		
Community identity		64.3 ± 19.1
Feel common bond to community		17.5 ± 5.3
Feel common identity to community		16.4 ± 5.5
Commitment to community		13.9 ± 4.4
Willingness to attend community health program		
Yes	351 (45.4)	
No	328 (42.4)	
Not sure	95 (12.2)	

We asked the participants to score the importance of community health issues and proposed actions to address them using a Likert-type scale ranging from 1 (*not important*) to 5 (*very important*). A score was calculated for each item, and these scores were ranked for each age group (Table 3). For all age groups, diseases-related health issues (cancer, cerebrovascular disease, and lifestyle-related diseases) were considered of high importance. In terms of proposed action, the participants expected that improving health services and access to medical checkups, opening vacant lots as public spaces for exercise, and maintaining collaborations with the community health office would improve community members’ health.

**Table 3:** Priority ranks for health problems and proposed action

Health Issue	Score (rank) by age group			Proposed Action	Score (rank) by age group		
	Young adults	Middle-age	Older adults		Young adults	Middle-age	Older adults
Cancer	190 (1)	1242 (1)	1428 (1)	Integrated medical check-up in one facility	194 (1)	1130 (1)	1269 (2)
Lifestyle-related diseases (metabolic syndrome, obesity, hypertension, diabetes mellitus)	179 (3)	1220 (2)	1292 (3)				
				Affordable and accessible medical check-up	188 (2)	1130 (1)	1220 (3)
Cerebrovascular disease	179 (3)	1148 (3)	1413 (2)	Community bus	151	1015 (2)	1279 (1)
Cardiovascular disease	159	1058 (5)	1247 (4)	Collaboration with community health office	157	983 (3)	1177 (4)
Musculoskeletal disease	137	951	1022				
Mental health problems	159	938	951	Utilizing open space as exercise place	183 (3)	974 (4)	1050
Sedentary lifestyle	180 (2)	1029	1123	Local farmers market	165 (4)	953 (5)	969
Unhealthy diet	159	916	1005	Exercise class and sports events	163 (5)	937	1047
Alcoholism	120	693	701	Nutritional education and healthy cooking class	146	879	995
Smoking	110	618	641				
Lack of health services and access to medical facilities	165 (5)	1064 (4)	1178 (5)	Agriculture class	133	953 (5)	969
				Smoking cessation program	119	755	799
Lack of public facility to exercise	169 (4)	977	1085	Role model and health leader	140	872	1011
Lack of information	156	941	1082	Inter-generation meet up	150	819	958
Low medical check-up rate	147	926	1033	Improving information dissemination	160	943	1135 (5)

*Numbers in parentheses indicate the top five highest priority scores. Some items received same total score and therefore shared the same rank*

Similar proportions of community members were willing and unwilling to participate in a community health program, and willingness to participate was highest among those aged 65 years or older. We asked the respondents to provide their reasons for not being willing to participate in such a program, and the most frequently mentioned answers were work-related (e.g., not having enough time because work is busy, being too tired from working on weekdays and wanting to rest on weekends, and thinking of participating after retirement). Other reasons listed for not being willing to participate in a community health program were being busy as caregiver for older family members or children, being unwilling to participate in a group (e.g., wanting to participate at their own pace, wanting to avoid trouble with other people, and feeling shy in front of other people), and having no friends of the same age with whom they could participate in such a program.

We used logistic regression analysis to determine which of the respondents’ characteristic was associated with their willingness to participate in a community health program. This analysis indicated that women, those with moderate-to-frequent past participation in community activities, those with a higher level of preventive behaviors, and those with a high commitment to the community had a relatively high odds of being willing to participate in a community health program (Table 4).

**Table 4:** Regression model for the determinants of willingness to participate in a community health program^a^

Predictor Variables^**b**^	β	SE of β	OR	95% CI of OR
Female	0.47	0.19	1.61	1.09 to 2.37
Unemployed	- 0.26	0.23	0.78	0.49 to 1.21
Education				
Elementary school/ Junior high	reference			
Senior high	- 0.38	0.35	0.68	0.34 to 1.36
College/ University	- 0.76	0.37	0.47	0.22 to 0.95
Age Group				
Young adults	reference			
Middle-age	- 0.02	0.36	0.98	0.48 to 2.00
Older adults	0.42	0.39	1.53	0.71 to 3.34
Participation in community activities				
Never/ seldom	reference			
Sometimes	1.13	0.34	3.11	1.63 to 6.20
Frequent	1.14	0.25	3.13	1.95 to 5.14
Preventive behaviors				
Low	reference			
Intermediate	0.33	0.22	1.39	0.91 to 2.12
High	0.66	0.26	1.94	1.16 to 3.26
Consider disease prevention as important	0.22	1.13	1.25	0.12 to 13.29
High common bond to community	- 0.11	0.23	0.89	0.57 to 1.41
High common identity to community	0.19	0.23	1.21	0.76 to 1.91
High commitment to community	0.65	0.23	1.92	1.23 to 3.02

Test: Akaike information criterion = 704, Nagelkerke pseudo-R^2^ = 0.19

There was no multicollinearity between the predictor variables

^a^ Criterion variable: Willingness to participate in community health program (yes/no)

^b^ Predictor variables (full model): age group, sex, educational background, employment status, importance of disease prevention, preventive behaviors, participation in community activities, community identity (common bond to community, common identity to community), commitment to community

### Priority decision and program development

The CAB assessed the priority of community health needs based on the results of the quantitative and qualitative data. The respondents to the questionnaire survey chose cancer as the top priority health issue, and they consequently chose the improvement of medical checkup facilities and increased access to medical facilities as the first proposed action. The participants in the group discussion mentioned the problem of the lack of a hospital that provides all the types of cancer screenings covered by the national program (i.e., screening for lung cancer, gastric cancer, colorectal cancer, prostate cancer, breast cancer, and cervical cancer). According to Nabari City data, there are two hospitals and 64 clinics in the city [35]. Among these facilities, 34 provide cancer screening services for city residents, but none provide all types of cancer screenings [36]. In the community we worked with in the present study, there was only one clinic that provided screenings for prostate cancer, lung cancer, and colorectal cancer. The community member participants therefore requested that a local medical facility provide integrated cancer screening services so that they would not have to travel to several medical facilities for cancer screening. In addition to the lack of service availability, public transportation to access medical facilities located in other parts of the city was also lacking. Among the participants in our study, those who were middle-aged or older were especially interested in having a community bus for easier access to these medical facilities. However, the city already conducts a mobile integrated cancer screening program in several types of locations, such as community centers, public health center, and city office [37]. Therefore, it appears that information about this program has not been well circulated among the community residents. Although we can advocate this type of change, upgrading the medical facilities was beyond our direct reach. Seeking to improve the dissemination of health information, including information about the mobile cancer screening program, was judged to be more feasible.

Despite lifestyle-related diseases ranking second in the questionnaire survey results, interest in this health issue was quite high among the qualitative study participants. Taking feasibility into account, the CAB decided to address lifestyle-related diseases as a health issue that they could work on with direct health and social outcomes, as well as intermediate health promotion outcomes that could be evaluated in a relatively short time period.

Taking the results from the quantitative study into consideration, the CAB decided to take advantage of the local resources and centered the program on three closely related themes considered highly relevant for daily life: health awareness, exercise, and nutrition. The CAB was divided into three smaller subgroups, each of which was responsible for developing ideas for one of these themes. Each of these subgroups had CAB members representing the three elements of the larger CAB: community members, university researchers, and city officials (public health nurses or community health office staff members). After a month of developing ideas for the program, each subgroup presented the results of their discussions in a CAB meeting to receive feedback from the other CAB members, and the full CAB then decided on the final program.

For the health awareness theme, health education classes were scheduled to raise awareness and knowledge. The health awareness CAB subgroup first identified community practitioners whom they wanted to ask for cooperation. Community practitioners were considered the best option for collaborators because these individuals have many interactions with community members and have gained their trust. These individuals were therefore invited to give educational talks for the community members. With lifestyle-related diseases as the main frame, the health awareness CAB subgroup met with each practitioner to decide on topics that were relevant to each individual’s expertise to be covered in their talks. Talks on six topics were planned in the following order:

1. Know your body: How to interpret the results of your health checkup
2. Healthy life expectancy and how exercise and nutrition contribute to it
3. Understanding lifestyle-related diseases and the importance of having a family doctor
4. From mouth to health (oral health)
5. Keeping your respiratory system healthy: from pneumonia to cancer, and preventive behaviors
6. Chronic disease management with lifestyle and appropriate cancer screening

The nutrition CAB subgroup considered the community members’ perspectives identified in the qualitative study results and generated a variety of nutrition intervention ideas, including organizing healthy cooking classes, establishing a community cafeteria with local ingredients from local farmers, opening a farmers’ market to offer fresh foods at low prices and to promote local products, and collaborating with local shops and eateries to provide nutritional information on their products and develop healthy menus. These ideas were presented at a meeting of the full CAB, who considered the available CAB resources and time limitations and decided to begin with conducting a healthy cooking class, opening a community cafeteria, and organizing a farmers’ market every 3 months. A registered dietitian in the community collaborated with the CAB nutrition subgroup to plan the cooking and nutrition education class.

The exercise CAB subgroup planned to optimize existing activities in the community, such as the square-stepping exercise classes that targeted older residents, seeking to improve their physical health and prevent dementia. Prior to the project, this activity was offered infrequently and had just started in a few areas of the community. Through the project, it was planned to expand these classes to more areas and to offer them more frequently (monthly). Because nature was considered a community strength by the study participants (see Table 1), a group walk around the area was planned. A *Hanami* walk, combining walking along a planned route with the Japanese custom of *Hanami*, enjoying the scenic spring while sitting under *sakura* (cherry blossom) trees. We also planned an exercise class in collaboration with an exercise instructor who lived in the community. We selected instruction on how to correctly walk for exercise and simple exercises that most people can do at home as the topics of the exercise class, covering highly accessible, basic exercises that different populations in the community can do at any time. Finally, to encourage the use of a recently opened community lot, a community *undokai* (sports festival) was planned. Unlike typical Japanese sports festivals, this festival was designed to include several measurements of physical strength. The festival thus served as a means of measuring community members’ physical health. Many of these activities were planned to be held on weekends to facilitate the attendance of those who were employed.

The university researcher CAB members took primary responsibility for designing the program evaluation. First, to assess the direct health outcome of the program, we planned to conduct physical measurements before, during, and after the interventions. The measurements also aimed to raise participants’ awareness in regard to their health status. We also planned to administer a questionnaire measuring knowledge (health literacy) three times over the course of the project. Because the community health leaders in the CAB had been trained by the city to use the “daily dietary check book” [38], we decided to use the same tool to record the participants’ food intake. We developed a health diary based on the one used by the Ministry of Health, Labour and Welfare [39], with an added section to record exercise, health measurement results, goals, and target achievement evaluations. All evaluation methods were presented in a CAB meeting and revised following feedback from the full CAB. Altogether, our project was named “Healthy Akame” project.

## Discussions

### Priority health issue

The participants in both the group discussion and the questionnaire survey prioritized diseases as a health problem of concern. Several diseases were mentioned, but cancer was the highest priority for all age groups. Cancer is the number one cause of death in Japan and also a major contributor to long-term care needs [2]. Various campaigns about cancer had previously been conducted in the community, so cancer was expected to be a concern for the community members.

Lifestyle-related diseases (i.e., metabolic syndrome, obesity, hypertension, and diabetes mellitus) and cerebrovascular diseases ranked second and third, respectively, in terms of their priority scores. For the middle-aged participants, lifestyle-related diseases ranked higher than cerebrovascular diseases, whereas cerebrovascular diseases ranked higher than lifestyle-related diseases for older adults. This result reflects the age at onset of these diseases. Type 2 diabetes mellitus and hypertension generally occur beginning in middle-age, and sometimes even in young adulthood [40, 41]. In contrast, the average age at onset of cerebrovascular disease is in the later adulthood, meaning that cerebrovascular disease affects more older adults than middle-aged or young adults [42]. This finding indicates that people’s health concerns and decisions regarding the prioritization of health problems reflect the epidemiology of diseases that may have affected the respondents or their family members. Likewise, a needs assessment conducted in the United States found that the top three health problem priorities in a studied community were consistent with the leading causes of mortality and morbidity nationwide [43].

Although the participants in the group discussion emphasized problems linked to unhealthy behaviors, unhealthy behaviors were not in the top five priorities identified in the questionnaire survey results. Furthermore, it was surprising that, despite lifestyle-related diseases being highly ranked for all age groups, unhealthy behaviors (which cause lifestyle-related diseases) were not considered important. One plausible explanation for these findings is that, for most people in the studied community, the concept of health centered on the absence or existence of diseases, leading to a lack of recognition of unhealthy behaviors as risk factors for diseases. In a study in Japan on knowledge about risk factors for cancer, a majority of the respondents were found to believe that the strongest risk factor for cancer was infection, rather than lifestyle-related risk factors [44]. In the present study, those who participated in the community forum group discussions might have been more likely than those who did not participate in the forum to have higher awareness about health and to emphasize the importance of the problem of unhealthy behaviors. A second plausible explanation for the difference between the qualitative and quantitative findings is that the data collection method (group discussion vs. questionnaire survey) may have affected the participants’ answers because of a difference in the anonymity of the responses.

Amidst the lack of recognition of unhealthy behavior as an important health challenge, in both the qualitative and the quantitative components of our study, young adult participants expressed concerns about sedentary lifestyles and the lack of public facilities for exercise. This finding reflects a growing awareness of the importance of physical activity and an unmet need to be physically active among young people. Clearly, this need should be addressed.

Smoking and alcohol drinking were the least prioritized unhealthy behaviors among all age groups. The results for the survey items gauging preventive behaviors show that only around 58% of the participants refrained from smoking, and an even smaller percentage abstained from drinking alcohol (33.8%). This means that large numbers of the participants drank alcohol and smoked. In fact, the percentage of smokers in this study was higher than Japan’s national average of 17.8% of the adult population who are smokers [45]. Our results are in line with Iwasaki et al.’s finding that more middle-aged men living in rural areas smoked and were heavy consumers of alcohol, compared with their counterparts living in urban areas [46].

Japan has enacted several policies on smoking, resulting in a decrease in the percentage of adult smokers from 21.8% (male: 36.8%, female: 9.1%) in 2008 to 17.8% (male: 29%, female: 9.1%) in 2018 [47]. However, the percentage of the total population who are daily smokers remains high [48]. Over the past decade, the percentage of the population reporting smoking in enclosed spaces (e.g., schools, offices, and restaurants) has decreased significantly, but the percentage reporting smoking in open spaces (e.g, public transportation areas, children’s playgrounds, and on the street) has not declined [47]. Compared with other Organization for Economic Co-operation and Development countries, Japan’s anti-smoking policies are still lacking, and further measures are necessary to foster a smoke-free environment [45]. In addition, alcohol consumption is common in daily life in Japan, and many social meetings are accompanied by *nomikai* (drinking party) [49–52]. Because smoking and drinking alcohol are part of social life in Japan, it may be difficult for people to perceive these behaviors as problematic. Changing the social norms of tobacco smoking and alcohol consumption in a society requires a population-level solution through stricter governmental policies and program enforcement. As we decided to take a disease-based approach with the theme of lifestyle-related diseases in the present study, we expected that our education program would be able to serve as an entry point to raise awareness about healthy and unhealthy behaviors, including smoking and alcohol consumption.

Previous studies conducting needs assessments in the general population also found that chronic diseases, such as cardiovascular and cerebrovascular diseases, were commonly identified as priority concerns through questionnaire surveys [17, 43, 53]. In contrast, when needs assessments were conducted using interviews or group discussions, more social determinants of health were identified as the top concerns, including environmental problems, financial problems, unemployment, unhealthy habits, and group-specific health problems (e.g., drug misuse among younger people and social isolation among older adults) [53].

Rural health needs assessment studies conducted in other countries have identified social determinants of health such as poverty [53, 54], unemployment, the difficulty of affording health care [18], and inequity in medical insurance coverage [43] as prominent issues. However, we did not find such issues in our study, possibly because, first, Japanese people have a high level pride and are reluctant to talk about personal financial issues. Many cases of poverty in Japan are undetected because people try to maintain a certain social appearance, or they themselves may not realize that they have been living under the poverty line [55, 56]. Second, Japan’s universal health insurance covers all citizens of the country, allowing them to access the medical care they need at any time and in any place [57]. This coverage is based on the social insurance system, which provides a public subsidy to ensure that the health needs of every citizen are covered. Therefore, the affordability of health care and inequity in health insurance were not areas of concern in the rural community in Japan that was the focus of the present study. However, this finding might also reflect that the minority groups that exist among rural citizens in Japan were not represented among the participants in our study.

### Plan of action

After selecting lifestyle-related diseases as the priority problem, the CAB developed a program to address this issue. The involvement of community partners in the CAB allowed us to identify the community’s resources and optimize their utilization to promote community health.

Focusing on three themes (health awareness, nutrition, and exercise), the CAB developed programs that were community-centered and appropriate. The health education classes were designed to raise awareness on various lifestyle-related diseases and to provide information on healthy behaviors that can prevent disease. In addition to providing knowledge, nutritional and exercise programs were designed with the aim of facilitating collective behavior changes. These programs were in line with the “Health Japan 21” strategy enacted by the Japanese government. Integrating this overall strategy with the local strengths (e.g., local farming and nature) and culture (e.g., *Hanami* walks) was expected to promote better uptake of the program in the community. The use of nature is also supported by previous findings: Maller et al. have summarized the health benefits of contact with nature, including relieving stress, improving well-being, and eliciting a parasympathetic nervous system response, which is associated with the restoration of physical energy [58].

The initiation of exercise classes and sports events and the use of a recently established open space for exercise were identified as very important interventions, both in the group discussion and in the questionnaire survey. These measures also address the abovementioned unmet need of young people in the community for physical activity. The promotion of physical activity among younger people can reduce the risk of disease in later life [59, 60] and open the door for attracting young people to participate in health promotion activities.

In addition to diseases and health behaviors, improving communication and information dissemination in the community were emphasized in the group discussion. Although other communication platforms were discussed, the CAB decided to use the existing community newsletter as the main communication method because most of the community participants were older adults, who might be more comfortable with a familiar method of information dissemination. However, this type of print communication requires community members to actively seek out information. If community members do not actively look for health information, the newsletter will be left unseen, and the information will not be transferred to the target. Other forms of communication, such as intergenerational communication and social events, were described as highly desirable by the group discussion participants, and several previous studies have shown the benefits of intergenerational activity for expanding social networks and improving health and quality of life [61–63]. However, intergenerational activity was not considered very important by most of the community residents who responded to our questionnaire survey. The group discussion method used for the qualitative component of our study may have brought the issue of intergenerational relations to light by prompting the discussion participants to experience a sense of togetherness. The self-administered questionnaire, in contrast, was anonymous, which may have allowed the respondents to be more honest in expressing the idea that intergenerational communication was not very important to them.

### Determinants of participation in community health program

We found that women were more likely to be willing to participate in a community health program. In the studied community, women are often more willing to participate and are more active in community activities in general, compared with men. Especially in rural areas, many older women enjoy socializing and participating in community activities, and this finding appears to be consistent across multiple countries. A recently published study conducted in Canada showed that, compared with rural men, more rural women wanted to participate in community activities and that the men more often reported being too busy to participate [64]. Women in Japan often take on the major responsibility for caring for the home and their family members [65]. Because of the nature of this role, these women may be more interested in health information, not only for their own health but also that of their family members, as has been shown in a Finnish study conducted by Ek [66]. In the present study, people who were strongly committed to the community were found to be more willing to participate in a future community health program. These people had a history of frequent participation in community activities. Regardless the type of activity, people who were committed to their community were willing to participate and to contribute to community programs.

Whereas people who practiced healthier behaviors were more likely to be willing to participate in a community health program, those with higher levels of education (college/university graduates) were less likely to be willing to participate. In the current era of unlimited information, people can access health information easily with the assistance of technology, and this may be especially true for those with relatively high levels of education. Jansen et al. have shown that people with higher levels of education might also have better health literacy, compared with those with lower levels of education [67]. This means that people with high levels of education might have a relatively good understanding of health information and of using health care services and resources; they might thus find it less important to participate in community health programs.

### Limitations

The participants in this study were mostly older adults, and only a few of the participants were young adults. The opinions obtained in this study therefore might not reflect the needs of younger people, who were a target of the community health program. Additionally, we did not actively involve local medical professionals of the community as part of CAB in this study. Although community needs and opinions regarding a plan of action are important, it is also necessary to consider the opinions of professionals who provide medical services to the people in the community. In Japan’s healthcare system, there is a barrier between the medical sector and the public health sector. From the layperson’s perspective, medical professionals are seen as occupying a very high position, and the community partners in our CAB were reluctant to burden busy medical professionals. For better collaboration in future initiatives, the active involvement of medical professionals is essential for designing and implementing efforts to improve the health of community members. Every community has its own unique characteristics, different resources, and varied support systems. These differences might result in different outcomes from those observed in our study. However, the research methods and approach that used in the present study can be applied in any community, with adjustment for the specific community conditions and context.

## Conclusions

The main health concern in our target community was identified as diseases. Although the results from the qualitative component of the study identified unhealthy behaviors as an important health issue, the results from the quantitative component of the study, conducted among a larger group of respondents, indicated that unhealthy behaviors were not considered very important to address as a health issue. Women, people who had previously participated in community activities, those who practiced preventive behaviors, and those with a strong commitment to the community were more likely to be willing to participate in a community health program.

Our findings demonstrated that the health priorities in the studied community in rural Japan were related to the epidemiology of diseases in the community. This study also showed that, despite a lack of awareness regarding unhealthy behaviors, there was growing concern among young adults in the community about sedentary lifestyles.

Involving community representatives as CAB members may have given us better insight into the community because these individuals had a good understanding of the community conditions. This approach enabled us to design a community-based health program that was tailored to the community members’ interests and to the unique local strengths and available resources.

## Supplementary Information

**Additional file 1.**

## Data Availability

The dataset supporting the conclusions of this article will be made available upon approval from the research CAB. Please contact the corresponding author to request access to these data.

## Abbreviations

CAB: Community advisory board
CBPR: Community-based participatory research
