# Supplementary material for: The “Healthy Akame!” community – government – university collaboration for health: a community-based participatory mixed-method approach to address health issue in rural Japan
Source: BMC Health Serv Res. 2020 Nov 30;20:1100. doi: 10.1186/s12913-020-05916-w (PMC7702688; doi:10.1186/s12913-020-05916-w)
Supplement: Supplementary file 1 — Additional file 1. [file 12913_2020_5916_MOESM1_ESM.pdf]

AKAME World Café Discussion Guide

Preface

Thank you for your cooperation as facilitator in the World Café discussion today.

Based on today's discussion, we will create a questionnaire to distribute to all Akame residents. We hope that the opinion received in this discussion forum and the following questionnaire survey can be utilized as a basis for thinking and planning community health activities in the future.

As part of the research process, written explanation about the research has been distributed to all participants and we have obtained the written informed consent. The world café moderator will explain about the background, purpose, general rules, and discussion flow of the world café to all participants before the group discussion started. The whole discussion process will be recorded with audio recording device for each group.

Please take consideration of the following grand rules for facilitators and facilitate the group discussion according to this guide.

Grand rules

1. There is no correct or wrong answer in this discussion. Each participant has the freedom to express their opinion.
2. We are interested in each of the participant's opinion. Regardless you agree or disagree with the opinion, please be understanding and listen to every opinion openly.
3. Conflicting opinions may occur. In the event of conflicts, please act as facilitator to ease the tension and prevent participants from arguing and fighting each other.
4. Please follow the main questions in this guide for each session. You can use the follow up questions and probes to elaborate answers from the participants. You may ask follow up questions other than those in the guide, when you deem it appropriate and necessary for the discussion.
5. If any difficulty or problem arise during the discussion that you cannot manage alone, please do not hesitate to ask help from the moderator or other CAB members.

Group discussion

This world café will be divided into 2 sessions of group discussion. Each of the group discussion is allotted 35 minutes (first 5 minutes for participants' introduction and the rest of it for the discussion). The world café moderator will notify in the remaining 10 minutes of each session.

Please facilitate the group discussion with the following flow.

1. Open the session by greeting participants and thank them for coming.
2. Introduce facilitator and their role in the group discussion.

3. Ask participants to introduce themselves briefly.
4. Ask the following main questions for each session.
   a. Session 1:
      "What is the main health issue that you consider important in your daily life as part of this community?"
   b. Session 2:
      "What are Akame community's strengths that are beneficial for health? How do you think they can contribute to resolving the health issues?"
5. Ask participants to first write their opinion keywords in the group paper, then explain their keywords.
6. Some participants may find it difficult to express their opinion, and sometimes the opinion exchange among participants does not happen smoothly. It may be easier to proceed by asking participants the following examples and follow-up questions.
   a. Session 1
      - Probes – Examples: mother and child health, older adults' health, diabetes, hypertension, sedentary lifestyle, etc.
      - Follow up – Why do you think it is important? Are there any other issues? How do the other participants think about the issue?
   b. Session 2
      - Probes – Examples: community sports club, reliable community health office staff, strong community bond, national health insurance, community clinic/hospital, etc.
      - Follow up – In what way can it help resolving the health issues? Has it been utilized before? Do you know such activities have been implemented in the community? What do you think about the existing community health activities? What are the strengths of the current activities that should be preserved? What kind of activities or program do you wish to have in the community?
7. Summarize discussion contents of each session briefly, ask the participants opinion regarding the summary.
8. End the session by appreciating participants for sharing their opinion and participating in the discussion process.
9. In the end of session 2, prepare for a 5 minutes' presentation. Choose one representative for the group to share the group discussion results in front of all world café participants.

Thank you
